# AGING AND CIRCADIAN CLOCK

**DOI:** 10.1093/geroni/igad104.1677

**Published:** 2023-12-21

**Authors:** Jennifer Hurley

## Abstract

Circadian rhythms are highly conserved, 24-hour, oscillations that tune physiology to the day/night cycle, enhancing fitness by ensuring that appropriate activities occur at biologically advantageous times. Disruption of proper circadian timing negatively impacts organismal fitness, making understanding the mechanism underlying circadian regulation over cellular physiology critical to appreciating a fundamental rule of life on earth. As we age, our bodies circadian rhythms change due to stress, chronic disruption of our clocks, neurodegeneration, and a host of other reasons, which can have a profound effect on our systems. Therefore, understanding the aging circadian clock is important to promote longevity and healthy aging. In this session, we will investigate some of the research going on that links the clock to aging. Topics will include the investigation of the role of the clock in timing immunometabolic regulation in the context of inflammation and Alzheimer’s disease, the optimization of the timing of exercise in the effort to maintain homeostasis and decrease risk, the connection between aging and the reduction of the number of rhythmically expressed genes and the weakening of circadian control, and the effect of dietary restriction on the circadian clock. The take home message of this session will be the importance of factoring daily time into research, preventative measures, and treatment regimens, to maximize overall health as we age.

